# Biologically adaptive radiotherapy for oropharyngeal Cancer (BARitOne): An R-IDEAL stage 2a-2b technical optimisation, feasibility, and safety study – Study protocol

**DOI:** 10.1016/j.ctro.2026.101245

**Published:** 2026-07-25

**Authors:** S. Anderson, M.C. Douglas, R. Valentine, L. Grocutt, G.J. Inman, L. Hay, S. Wright, K. Mair, C. Kelly, R. Seddon, A. Shaw, MacMillan A, J. Gourlay, J. Flach, E. Au, C. Vens, C. Schoenherr, D. Grose, C. Lamb, S. Schipani, S. Vohra, C. Wilson, C. Paterson

**Affiliations:** a Beatson West of Scotland Cancer Centre, Glasgow; b Queen Elizabeth University Hospital, Glasgow; c Glasgow Royal Infirmary, Glasgow; d CRUK Scotland Institute, University of Glasgow, Glasgow; e Glasgow Oncology Clinical Trials Unit, School of Cancer Sciences, University of Glasgow, Glasgow, UK; f School of Cancer Sciences, Wolfson Wohl cancer centre, University of Glasgow, Glasgow, United Kingdom; g Glasgow Head & Neck cancer (GlaHNC) research group, CRUK RadNet Glasgow+, United Kingdom; h Patient and public involvement representative, United Kingdom

**Keywords:** biologically adaptive, radiotherapy, head and neck cancer, dose escalation

## Abstract

**Background:**

The incidence of head and neck squamous cell cancer (HNSCC), including HPV-positive and negative oropharyngeal squamous cell cancer (OPSCC) is rising. HPV-negative and some HPV-positive OPSCC has poor outcomes. Attempts to uniformly intensify treatment in poor-prognosis HNSCC populations have been unsuccessful. Diffusion-weighted MRI (DW-MRI) has been used during radiotherapy to identify OPSCC which will fail standard radiotherapy. Biologically-adaptive, DW-MRI guided radiotherapy may allow dose-escalated radiotherapy to be delivered to populations predicted to fail standard treatment, i.e. those really in need of treatment intensification. While individual components of response-adapted workflow are not novel, their combination is significantly different. The BARitOne (**Biologically Adaptive Radiotherapy for Oropharyngeal Cancer)** study aims to allow optimisation and evaluate feasibility of this approach.

**Methods:**

BARitOne is a single-centre R-IDEAL stage 2a and b study of patients with poor-prognosis stage 3-4b OPSCC undergoing single-modality radical radiotherapy. All participants commence standard radiotherapy, 65Gy in 30 fractions. DW-MRI at fraction 1 and 10 assess change in apparent diffusion coefficient (ADC) in primary and nodal disease and stratify tumours into ‘responders’ and ‘non-responders’. Non-responders receive dose-escalation (40.5Gy in 15 fractions) to the gross tumour volume (GTV) for fractions 16 to 30;total dose 73Gy in 30 fractions to GTV. Responders continue standard treatment. The primary aim is to evaluate the proportion of participants that complete DW-MRI guided response-adapted radiotherapy.

**Discussion:**

Identifying non-responding tumours is an essential step towards individualising intensified radiotherapy for high-risk disease. The additional burden that image-guided, response-adaptive radiotherapy brings, along with the imperative to avoid gaps in radical treatment means workflow needs development and optimised prior to large-scale implementation and comparison to the current standard. BARitOne offers this, with learning also transferrable to other tumour types. While uniform treatment intensification for entire poor-prognostic HNSCC populations has failed to improve survival outcomes, this individualised and biomarker-guided approach to intensification offers an alternative strategy.

**Trial registration:**

International Standard Randomised Controlled Trial Number ISRCTN45653542.

## Highlights

• High risk oropharyngeal cancer is associated with a poor prognosis.
• Uniform radiotherapy intensification to high-risk patients did not improve outcomes.
• Using Diffusion Weighted-MRI as a biomarker of response may allow dose-escalation.
• BARitOne evaluates patient proportion that completes response-adapted radiotherapy.
• Grade 3 toxicity, patient quality of life, disease control are secondary endpoints.

## 1 Introduction and background

Head and neck cancer is the 9th most common cancer in the UK, with 12,800 new cases annually, and numbers expected to rise to 16,300 by 2040 [1]. Increasing numbers of patients with oropharyngeal squamous cell cancer (OPSCC) is not only due to the well documented growing incidence of the Human Papillomavirus (HPV), but also due to an increase in HPV-negative cancers [2]. Between 2010 and 2020, the rate of OPSCC increased by a factor of 2·1, and this is projected to more than double between 2020 and 2039 [3]. Survival is known to be favourable with HPV-positive disease, with a 3 year survival of 93%, leading to the consideration of possible treatment de-escalation [4], [5]. However, patients with HPV-negative cancers, and those with a substantial smoking history regardless of tumour HPV status, have poorer survival outcomes, with a 3 year overall survival as low as 46.2% [4], [6]. While HPV-negative and HPV-positive disease are different entities, prognosis is affected by smoking history in addition to tumour HPV status. Poorer outcomes, compared to the HPV-positive non-smoking population, is seen in both groups, with a common unmet need to improve disease control and survival. With a plethora of negative studies examining uniform de-intensification for HPV-positive OPSCC there is now acceptance that a more personalised approach to de-escalation of treatment is required [7], [8], [9], [10]. For example, promising results have been demonstrated in the Phase II setting using 18F- fluoromisonidazole Positron Emission Tomography (F-MISO PET) to guide hypoxia-directed dose de-escalated radiation [11]. Attempts to uniformly intensify treatment in the poorer prognosis populations have similarly been unsuccessful to date, both in the oropharyngeal [12] and non-oropharyngeal sub-sites [13]. An imaging biomarker to guide intensification of treatment for the poorer prognosis population remains an urgent unmet need.

Standard treatment for locoregionally advanced oropharyngeal cancer is 6–7 weeks of chemoradiotherapy with platinum-based chemotherapy [14]. Considering poorer outcomes, it is important that patients with HPV-negative OPSCC receive this standard of care treatment, however there are often associated factors that preclude the chemotherapy component. Socioeconomic deprivation and HPV-negative disease [15] are knowingly linked, as well as a history of smoking and/or alcohol, often leading to multimorbidity. These factors can affect performance status and suitability for concurrent treatment [16], [17], [18]. In addition, the benefit of cisplatin is not seen in patients aged 70 years old or older, and the average age at diagnosis with HPV-negative disease is 66 years [19]. Recent data has shown as many as 41% of patients are receiving single modality radiotherapy, despite having indications for concurrent chemotherapy [20]. UK guidelines recommend that concomitant cisplatin with curative radiotherapy is offered to patients who are ‘young and fit enough’ to tolerate the likely increased toxicity. [21]. Recent work evaluating fraily in the oropharynx cancer population in our region found that over half of patients were categorised as moderately or severely frail, [22] giving an insight to the challenges in delivering optimal treatment. This may contribute to poorer outcomes [23], [24], and highlights the clinical need for an alternative, intensified radiotherapy approach.

Intensifying an already morbid treatment regime, in a patient population that is often unsuitable for standard of care treatment, needs to be carefully considered. Yet, as the pattern of treatment failure is predominantly inadequate loco-regional control in both HPV-positive and negative disease, radiotherapy dose-escalation is an enticing option [4]. A dose-response relationship in head and neck squamous cell cancer (HNSCC) is recognised, however escalating dose clinically has revealed mixed results. Increasing dose can result in increased toxicity rates [25], with other studies showing frequency and severity of toxicities remaining similar [26]. Of note, these studies, along with the ones described earlier, have uniformly increased treatment to all poor prognosis patients. Intensifying treatment only for those who really need it, i.e. those tumours predicted to respond inadequately to standard treatment, allows the risk of increased toxicity to be limited to the sub-group most in need.

The dose received by organs at risk (OARs) underpins the likelihood of patients developing toxicities with radiotherapy. An increase in delivered dose raises concerns about increased doses received by the multiple OARs in close proximity to the target volumes. However, leveraging modern planning technology with both RapidPlan™ and multi-criteria optimisation (MCO), a prior in silicio study demonstrated that it was possible to increase the dose to the gross target volume (GTV) in OPSCC by 12% compared to standard of care, without increasing dose to either OARs or surrounding planning target volumes (PTV), considered a surrogate of adjacent mucosa [27]. In that study a mandatory constraint of no more than 1.75 cc of the primary gross tumour volume (GTVp) was allowed to receive > 84 Gy, this having previously been shown to be the dosimetric threshold above which late grade 4 mucosal ulcers will develop [28]. This constraint was met in all plans, and will be taken forward clinically in BARitOne to help mitigate the risk of increased toxicity at the primary tumour site.

MRI is the preferred imaging modality to accurately define the extent of OPSCC [21], [29]. Diffusion weighted MRI (DW-MRI) demonstrates the Brownian motion of water protons, also known as molecular diffusion. This can be quantified by the application of the apparent diffusion coefficient (ADC) which takes the microscopic motions of capillary perfusion and molecular diffusion into consideration, detailing the microstructure of the tissue of interest [30]. Previous studies have shown that ADC values can be applied to predict and monitor response to treatment [31], [32]. Solid masses hinder diffusion and have low ADC values. Several studies in HNSCC have shown that increases in ADC during treatment were significantly larger in tumours achieving a complete response [33], [34], [35], [36]. This specifically has been evaluated in poor prognosis OPSCC; an ADC assessment at baseline then fraction 10 of radiotherapy was found to appropriately categorise responding disease versus non-responding [37]. Primary disease with an increase in ADC of >17.8% was found to have a positive predictive value of 92.1% and a negative predictive value of 21.2% of long term disease control. This study mirrors findings from Wong et al.'s study, where a threshold ADC value of >17% and a nodal ADC of >27% was found to have positive predictive values of over 90% [38]. These studies suggest that serial DW-MRI may be used as an imaging biomarker to identify the sub-group of patients with poor prognosis OPSCC predicted to be failed by standard radiotherapy, and therefore may benefit from treatment intensification.

Adaptive radiotherapy is the process of adjusting the radiation plan during the course of treatment. This can be carried out with regards to anatomical changes, or response to treatment. Anatomical adaptive therapy accounts for the structural changes that can occur during treatment such as tumour reduction, weight loss or patient movement [39] and is commonplace during a radical treatment course. However, the concept of adaptive radiotherapy based on response assessment during treatment is not yet incorporated into clinical practice, but would allow individualisation of treatment.

In summary, patients with poor prognosis OPSCC are often unsuitable for standard of care concurrent chemoradiotherapy due to factors such as poor performance status or comorbidity. Uniform treatment intensification for this group has not resulted in better outcomes. Therefore survival remains poor, and an alternative regime that patients can tolerate is required. Predicate studies suggest that dose escalation with DW-MRI guided, response-adapted radiotherapy for poor prognosis OPSCC is now a feasible clinical possibility. However, the logistics and practicability of such a novel workflow need to be evaluated and optimised prior to larger scale comparison to the current standard. The BARitOne study tests the hypothesis that it is feasible to deliver DW-MRI guided response-adapted radiotherapy.

## 2 Methods/design

### 2.1 Trial design

Due to the nature of advances in radiotherapy, the standard phase 1, 2 and 3 trial frameworks, developed to evaluate new drugs, is not the most appropriate [40]. Instead, an approach in line with trials in surgical innovation is more suited [41], [42]. The IDEAL (Idea, Development, Exploration, Assessment and Long-term) study framework, developed for the assessment of surgical advances, has previously been tailored for radiotherapy; the R-IDEAL approach has been used, for example, to evaluate the clinical implementation of the MRI-guided linear accelerator (MR-linac) by the MR-linac consortium [43], [44].

BARitOne is a stage 2a (Development) and 2b (Exploration) single centre study of patients with poor prognosis stage 3-4b OPSCC planned for single modality radical radiotherapy. This study is sponsored by NHS Greater Glasgow and Clyde (sponsor reference GN210N536) and funded by the Beatson Cancer Charity (Grant reference 21, 22–035 BCC), ethical approval was granted (Research Ethics Committee reference: 23/WS/0056). Clinical study registration number is ISRCTN45653542.

### 2.2 Trial population

Patients are identified and assessed by the clinician coordinating their care and are eligible to enter the trial according to the criteria in Table 1. All of the inclusion and none of the exclusion criteria must be present for trial eligibility.

**Table 1:** – Inclusion and exclusion criteria for all patients in BARitOne trial.

Inclusion Criteria	Exclusion criteria
Age 18 years or older	Head and neck cancers from sub sites other than oropharynx
Histologically confirmed HPV-negative OPSCC or HPV-positive OPSCC and a significant smoking history of 10 pack years within 20 years of diagnosis	Planned for concurrent radiotherapy with chemotherapy or cetuximab
	HPV-positive OPSCC in patients with no significant smoking history (low risk OPSCC)
Stage 3, 4a or 4b	Confirmed distal metastatic disease (Stage 4c)
Scheduled for single modality photon radiotherapy as primary radical treatment	Patients with contra-indications to MRI scanning eg; claustrophobia, metallic foreign body, pacemaker/implant, allergy to gadolinium contrast, eGFR <30mls/min.
Ability to provide written informed consent prior to participating in the trial and any trial related procedures being performed	Patients who have undergone primary surgery for OPSCC (including neck dissection or received induction chemotherapy prior to definitive treatment
Patients willing and able to comply with protocol for duration of study	Patients receiving chemoradiotherapy or cetuximab-radiotherapy or has prior radiotherapy to the H&N region
	Patients who do not adequately understand verbal or written information

### 2.3 DW-MRI scans

Baseline MRIs are obtained immediately prior to fraction one (MRI_1) and after ten fractions of radiotherapy (MRI_2). Sequences include 3D T2, DW (b numbers 0, 50, 100, 500, and 1000: all are used to calculate the ADC map), t1 FS Post Contrast and t2 dixon. GTVp and GTVn are outlined on each ADC map using anatomical and functional sequences to inform delineation. ADC is measured for each target volume and the % change in ADC is calculated between MRI_1 and MRI_2. Tumours deemed to be responding inadequately to radiotherapy (‘non-responders’) are those with an increase in GTVp ADC <18%, or GTVn ADC <27%, the remainder categorised as ‘responders’. ADC measurements and the categorisation of responders versus non-responders are overseen by the trial safety committee, which includes the principal investigator, project manager, study statistician, trial co-ordinator, trial monitor and sponsor's representative.

### 2.4 Radiotherapy planning and treatment

Standard radiotherapy planning procedures are undertaken. For planning and treatment, patients are immobilised with a custom-made S-Frame thermoplastic mask, fixated to the Portrait overlay board or treatment couch insert (Oncology Imaging Systems – OIS). Patients shoulder position is maintained by holding hand grips attached to the board. Head and neck position is reproduced using an individualised foam neck rest placed on top of a Silverman headrest. Contrast enhanced CT and MRI planning scans are obtained. Target delineation is carried out by the treating oncologist, using all available clinical and radiological information from diagnosis and staging and with reference to international guidelines [45], [46]. Two clinical target volumes (CTVs) are created, CTV65, encompassing primary and nodal gross tumour volume (GTVp and GTVn respectively) with a 5-10 mm margin and CTV54 to include areas at high risk of microscopic disease. PTVs are created with an isotropic margin of 3 mm in all directions from each CTV. Peer review and approval of target volumes is mandatory and standard practice in our centre for all H&N cancers.

Radiotherapy treatment plans are created using the Eclipse™ treatment planning system (TPS) (Varian medical systems, Palo Alto, CA) and approved by the treating clinician. Dose constraints for target volmes and OARs are as shown in Table 2, and are based on standard institutional planning guidelines. Treatment plans are optimised to achieve all specified OAR constraints wherever possible, while maintaining protocol defined target volume coverage. Eclipse™ uses photon optimiser (PO) for plan optimisation and Acuros® XB advanced dose calculation algorithm v16.1 for dose calculation. All radiotherapy treatment plans are generated using a calculation grid size of 2.5 mm. All patients receive radiotherapy delivered on a Varian TrueBeam linear accelerator using Rapid Arc® (Varian Medical Systems, Palo Alto, CA). A 6MV photon energy, 600 MU/min dose rate and collimator jaw tracking are used for all volumetric-modulated arc therapy (VMAT) plans. Radiotherapy is delivered in thirty fractions, given daily Monday to Friday over six weeks. Daily on-line image guidance with CBCT and/or KV-KV imaging is utilised.

**Table 2:** – dose constraints for target volumes and OARs.

Structure	Constraint	
	Non-escalated	Escalated
GTV_All	*D99% > 90%*	*D99% > 90%*
	*D95% > 95%*	*D95% > 95%*
	*D50% ± 1Gy of prescribed dose*	*D50% ± 1Gy of prescribed dose*
	*D5% < 105%*	*D5% < 105%*
	*D2% < 107%*	*D2% < 107%*
PTV65	*D99% > 90%*	*D99% > 90%*
	*D95% > 95%*	*D95% > 95%*
	*D50% ± 1Gy of prescribed dose*	*D50% ± 1Gy of prescribed dose*
	*D5% < 105%*	*D5% < 110%*
	*D2% < 107%*	*D2% < 115%*
PTV54	*D99% > 90%*	*D99% > 90%*
	*D95% > 95%*	*D95% > 95%*
	*D50% ± 1Gy of prescribed dose*	*D50% ± 1Gy of prescribed dose*
	*D5% < 117%*	*D5% < 117%*
	*D2% < 122%*	*D2% < 122%*
SpinalCord_PRV	*Dmax < 48Gy*	*Dmax < 48Gy*
	*D1% < 44Gy*	*D1% < 44Gy*
Brainstem_PRV	*D1% < 48Gy*	*D1% < 48Gy*
Contralateral Parotid	*Dmean < 24Gy*	*Dmean < 24Gy*
Larynx	*Dmean < 40Gy*	*Dmean < 40Gy*
Mandible	*Dmax < 65Gy*	*Dmax < 65Gy*
Oesophagus	*Dmean < 40Gy*	*Dmean < 40Gy*
Trachea	*Dmean < 40 Gy*	*Dmean < 40 Gy*

For this study, two phases of treatment are planned and prescribed, each 32.5 Gy in 15 fractions. PTV65 receives 32.5Gy in 15 fractions in Phase I_plan 1 in all patients. In phase II, there are 2 options. Phase II_plan 1 is identical to Phase I_plan 1 and used for tumours assessed to be responding adequately to standard radiotherapy. An escalated plan for Phase II is also created, which involves delivering 32.5 Gy in 15 fractions to PTV65 but with a boost of 40.5 Gy in 15 fractions to GTVn and GTVp, Phase II_plan2. The decision for Phase II_plan1 or Phase II_plan 2 depends on the assessment of MRI_1 and MRI_2 as described above. All target volumes, in particular, GTVp and GTVn for Phase II are unchanged from baseline delineation. Dose to non-responding tumours is escalated for both GTVp and GTVn regardless of which is the non-responding component.

A modelling study suggests that increasing dose intensity halfway through treatment with a biphasic fractionation schedule may overcome the problem of tissue hypoxia, and tissue re‑oxygenation during treatment [47].

**Fig. 1:**
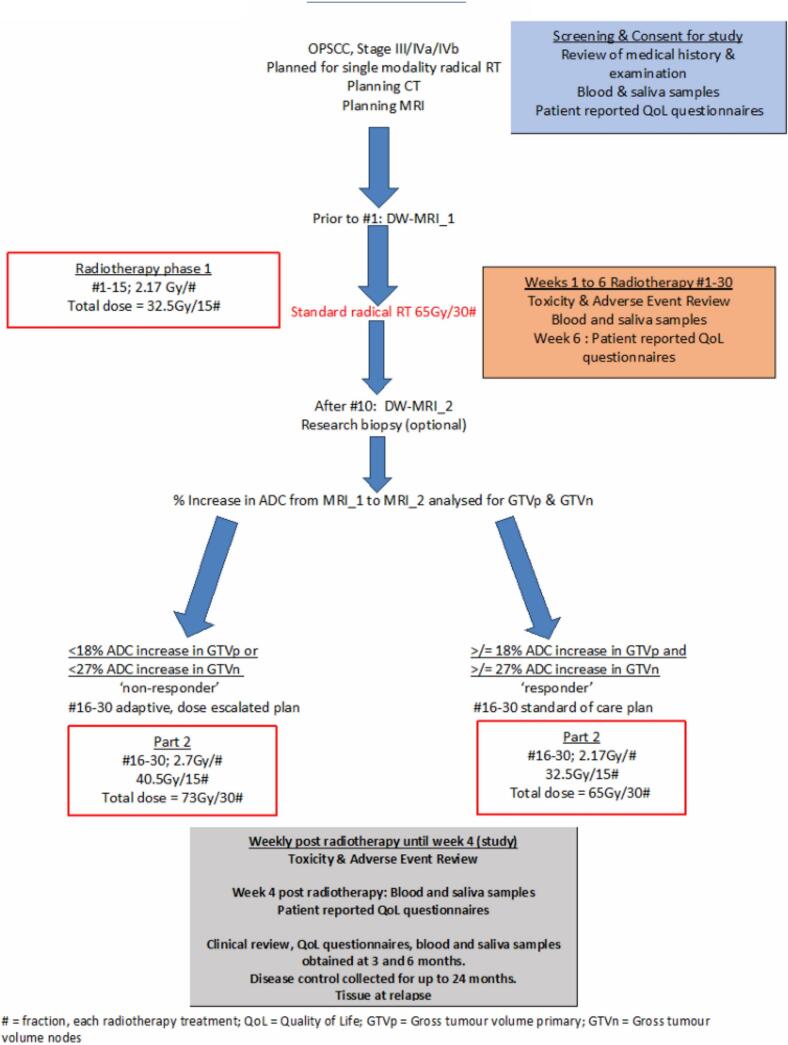
Trial schema.

The trial schema is visualised in Fig. 1.

### 2.5 Assessments and endpoints

The schedule of study assessments is detailed in Table 3 below. The following assessments are conducted at baseline and week 6 of radiotherapy, then at 4 weeks, 3 months and 6 months after radiotherapy completion: MD Anderson Dysphagia Inventory score (MDADI) and quality of life questionnaires (EORTC QLQ-C30 and H&N43). Adverse events will be assessed, using CTCAE v5.0 [48], at baseline and weekly during radiotherapy until 4 weeks post completion, then at 3 and 6 months post radiotherapy. An optional sub-study will collect blood and saliva samples at baseline, weekly during radiotherapy and at week 4, then 3 and 6 months post radiotherapy. A further optional procedure for participants is to undergo a research biopsy from the primary tumour after ten fractions of radiotherapy (after MRI_2).

1. CTCAE v5.0 will be used to grade acute toxicity, RTOG Radiation Morbidity Scoring Criteria will be used to grade late toxicity, at 3 & 6 months post completion of RT
2. Quality of Life questionnaires include EORTC QLQ-C30 & H&N43

**Table 3:** –Schedule of assessments.

Study assessment	Baseline	RT Week 1	RT Week 2	RT Week 3	RT Week 4	RT Week 5	RT Week 6	Post-RT Week 1	Post RT week 2	Post RT week 3	Post RT week 4	Post RT 3 monts	Post RT 6 months
Performance status ECOG	X						X				X	X	X
Swallowing assessment (MDADI)	X						X				X	X	X
Toxicity review^1^	X	X	X	X	X	X	X	X	X	X	X	X	X
QoL Questionaires^2^	X						X				X	X	X
Translational blood & saliva sample	X	X	X	X	X	X	X				X	X	X
Research biopsy				X									

Loco-regional control rates at 12 and 24 months, as well as overall survival will also be collected from record review. Safety reporting will be carried out by the Pharmacovigilance Department of the Glasgow Oncology Clinical Trials Unit.

**Primary Endpoint:**

The proportion of patients able to complete DW-MRI guided response-adapted radiotherapy with a target of 50%.

The benchmark of 50% was a pragmatic decision, selected to be permissive enough to avoid prematurely abandoning a potential new treatment strategy, yet with a predefined threshold to indicate futility of further evaluation. It allows for an important activity of both the study and the R-IDEAL framework, optimisation of the novel workflow.

**Secondary Endpoints:**

Proportion of Grade 3+ acute (up to 3 months) and early late (3–6 months) toxicity rates, comparing dose-escalated and standard radiotherapy cohorts.

Patient reported quality of life scores, comparing dose-escalated and standard radiotherapy cohorts.

Loco-regional control rates at 12 and 24 months, overall survival, and cancer specific survival; comparing dose-escalated and standard radiotherapy cohorts.

**Exploratory Endpoints:**

Safety and feasibility of research biopsy at #10 of radiotherapy.

Feasibility of molecular profiling studies (including panel-based/WES DNA sequencing, bulk RNA profiling and spatial analysis of RNA expression and protein on baseline and #10 biopsies.

Feasibility of identifying circulating biomarkers (including ctDNA, metabolites, circulating immune cell populations and cytokines/chemokines) from blood, plasma and/or saliva.

### 2.6 Statistics

A sample size of 20 patients will be targeted. Using a one-sided significance level of 0.05 and 80% power, the true success rate would need to be approximately 0.76, to demonstrate a success rate of greater than 50%. For the primary endpoint of feasibility, the proportion of patients able to complete DW-MRI guided response adapted radiotherapy will be reported with an exact 95% confidence interval. For the secondary safety analysis, the proportion of dose escalated patients experiencing grade III or IV RT-related toxicities will be compared with standard treatment patients using an odds-ratio with an exact 95% confidence interval. The time of onset and duration of severe toxicities, as well as the number of patients missing one or more treatment days, will also be reported. The number of patients achieving loco-regional control at 6-months follow-up will be reported, along with an odds ratio for the comparison of groups and an exact 95% confidence interval. Overall survival and cancer-specific survival will also be summarised at 6-months follow-up, presented with Kaplan-Meier plots and log -rank tests.

## 3 Discussion

Previous studies have shown that functional imaging with DW-MRI can be used to predict outcomes in head and neck cancer and identify those who are at risk of treatment failure [32], [33], [37], [49]. It has also been shown that dose escalation can improve outcomes in HNSCC, whilst maintaining similar toxicity and doses to OARs. However, in large scale randomised studies, uniform treatment intensification has not led to improved disease control. Identifying non-responders to radiotherapy is an essential step towards individualising treatment, allowing the delivery of patient specific intensified treatment to those who need it, whilst continuing current standard of care for those with adequately responding disease.

Standard of care OPSCC radiotherapy involves complex treatment planning with significant radiographer, clinician and medical physics input prior to it commencing. Once started, patients continue on this pre-determined treatment plan in all but exceptional cases, and without any evaluation of treatment response. Assessment of response tends to be carried out weeks-months after radiotherapy completion, with no opportunity to modify the already delivered treatment course. Adaptive radiotherapy, based on response to radiotherapy in real time, may allow an individualised approach to treatment but requires a novel and even more complex workflow than is standardly used. Additional imaging and its analysis, as well as the increased burden from adaptive planning and the time pressure to compete these steps while avoiding gaps in daily treatment is a potential barrier. This study will aim to address the feasibility of this in an already busy clinical department and optimise the steps required. This will facilitate further up-scaling to multi-centre and randomised studies evaluating biologically adaptive radiotherapy for poor prognosis OPSCC, with learning also being transferrable to other tumour types. While uniform treatment intensification for entire poor prognostic HNSCC populations has failed to improve survival outcomes [12], [13], this individualised and biomarker guided approach to intensification offers an alternative strategy.

## Abbreviations

**Table:** 

Abbreviation	Term
ADC	Apparent Diffusion Coefficient
CTU	Clinical Trials Unit
CTV	Clinical Treatment Volume
DW-MRI	Diffusion Weighted MRI
F-MISO PET	Fluoromisonidazole Positron Emission Tomography
GTV	Gross Tumour Volume
HPV	Human Papillomavirus
HNSCC	Head and Neck Squamous Cell Carcinomas
MCO	Multi-Criteria Optimisation
MDADI	MD Anderson Dysphagia Inventory score
OAR	Organ At Risk
OPSCC	Oropharyngeal Squamous Cell Carcinoma
PTV	Planning Treatment Volume
R-IDEAL	Radiotherapy Idea, Development, Exploration, Assessment, and Long-term study
RT	Radiotherapy

## Ethics approval and consent to participate.

The study will be conducted in line with the current Government, HRA and health board guidance. The trial will be carried out in accordance with the World Medical Association Declaration of Helsinki (1964) and its revisions (Tokyo [1975], Venice [1983], Hong Kong [1989], South Africa [1996] and Edinburgh [2000]). Favourable ethical opinion will be sought from an authorised REC before patients are entered onto this clinical trial. Research Ethics Committee reference: 23/WS/0056.

Signed participant consent to enter the trial will be sought from each participant only after full explanation has been given, an information sheet offered and time allowed for consideration. Participants have the right to refuse to participate without giving reasons, and alternative treatments remained an option to that specified in the protocol at any stage if in the best interests of the participant. All participants are free to withdraw at any time from the protocol treatment without giving reasons and without prejudicing further treatment. Original completed consent forms will be retained at each site, photocopied in the patient's medical records, and all patients will be given a copy. All patients will be re-consented if protocols are updated, and remote consents are permitted via phone or video.

## Consent for publication

Consent for publication has been granted by the BARitOne Trial Monitoring Group (TMG).

## Funding

10.13039/100009232Beatson Cancer Charity (Funding Application Number 20–21-034), CRUK RadNet Glasgow+ Centre (CRUK Award Number is: RRCOER-Jun24/100003).

## Declaration of competing interest

The authors declare that they have no known competing financial interests or personal relationships that could have appeared to influence the work reported in this paper.
